# Impaired type I interferon regulation in the blood transcriptome of recurrent asthma exacerbations

**DOI:** 10.1186/s12920-018-0340-3

**Published:** 2018-02-27

**Authors:** Jose L. Gomez, Maria P. Diaz, Gustavo Nino, Clemente J. Britto

**Affiliations:** 1 Division of Pulmonary, Critical Care & Sleep Medicine, Department of Internal Medicine, Yale University School of Medicine, New Haven, CT USA; 2 Division of Pulmonary and Sleep Medicine, Children’s National Medical Center, Washington, DC USA; 3 Department of Pediatrics, George Washington University School of Medicine and Health Sciences, Washington, DC USA; 4 Center for Genetic Medicine, Children’s National Medical Center, Washington, DC USA

**Keywords:** Asthma exacerbation, Transcriptome, Innate immunity, Antiviral immunity, Asthma, Infection

## Abstract

**Background:**

Asthma exacerbations are an important cause of morbidity in asthma. Respiratory infections are often involved in asthma exacerbations in both children and adults. Some individuals with asthma have increased susceptibility to viral infections and as a result increased rates of asthma exacerbations. We sought to identify a transcriptomic signature in the blood associated with asthma exacerbations triggered by respiratory infections (AETRI) and determine its association with increased risk for asthma exacerbations.

**Methods:**

We conducted a two-step study using publicly available, previously generated transcriptomic signatures in peripheral blood mononuclear cells (PBMCs) from asthmatics to identify novel markers of increased risk for asthma exacerbations. In the 1st step, we identified an in vitro PBMC signature in response to rhinovirus. In the 2nd step, we used the in vitro signature to filter PBMC transcripts in response to asthma exacerbations in an independent in vivo cohort. Three different subgroups were identified and studied in the in vivo cohort: 1. Single AETRI; 2. Multiple AETRIs; and 3. Single non-infectious asthma exacerbations. We performed pathway and network analyses in all independent comparisons. We also performed an immunologic gene set enrichment analysis (GSEA) of the comparison between single AETRI and non-infectious asthma exacerbations.

**Results:**

The in vitro signature identified 4354 differentially expressed genes (DEGs) with a fold change (FC) ≥ 1.2, false discovery rate (FDR) < 0.05. Subsequent analyses filtered by this in vitro signature on an independent cohort of adult asthma identified 238 DEGs (FC≥1.1, FDR < 0.1) in subjects with a single AETRI and no DEGs in single non-infectious asthma exacerbations. A comparison between the response in subjects with single and multiple AETRIs identified two discordant gene subsets. In the largest discordant subset (*n* = 63 genes) we identified an impaired type I interferon and *STAT1* response in multiple AETRIs during the acute phase of the exacerbation and an upregulated *STAT1* response at baseline. The *STAT1* upregulation at baseline in subjects with multiple AETRIs was accompanied by upregulation of pro-inflammatory molecules including IL-15, interferon-stimulated genes (ISGs), several toll-like receptors 2, − 4, − 5 and − 8 and a triggering receptor expressed on myeloid cells 1 (*TREM1*) network.

**Conclusions:**

Subjects with asthma and multiple AETRIs display a pro-inflammatory signature at baseline, associated with elevated *STAT,* IL-15 and ISGs, and an impaired *STAT1* response during acute asthma exacerbations.

**Electronic supplementary material:**

The online version of this article (10.1186/s12920-018-0340-3) contains supplementary material, which is available to authorized users.

## Background

Asthma exacerbations are associated with increased emergency visits and hospitalizations; in severe cases they can lead to respiratory failure and death. The incidence of asthma exacerbations has steadily risen over the last decade. The Centers for Disease Control National Surveillance of Asthma found that as of 2010, 13.9 million people experienced an exacerbation in the previous year, a 2.9 million increase since 2003 [1]. Exacerbations can be triggered by a variety of stimuli including respiratory infections, cigarette smoke, allergens, exercise, occupational exposures, and medications. Previous studies have shown that a history of a severe exacerbation is a risk factor for subsequent exacerbations [2]. However, the mechanisms by which these triggers precipitate an exacerbation and those that are associated with recurrence are incompletely understood. Therefore, a unified approach to asthma exacerbations based on biologic risk factors and clinical features would improve our ability to stratify individuals at risk for recurrent exacerbations and provide novel therapeutic targets.

Respiratory infections are a common specific trigger of exacerbations with estimates in the 80% range for both children and adults; rhinoviruses are the most frequently isolated pathogen [3, 4]. Several studies have shown that asthmatics have an impaired antiviral response that increases their susceptibility to viral infections and makes them prone to asthma exacerbations triggered by respiratory infection (AETRI). The mechanisms underlying this increased susceptibility include a deficient production of type I Interferons (IFNs), specifically IFN-α and IFN-β. Type I IFNs are involved in the antiviral response through activation of immune cells (natural killer and macrophages) and enhancement of antigen presentation through increased expression of major histocompatibility complex (MHC) and differentiation of virus-specific cytotoxic T lymphocytes [5, 6]. In asthmatics, although epithelial expression of type I IFNs during inflammation may be low, airway epithelial production of type I IFNs is impaired following exposure to rhinovirus [7]. This impairment is also present in alveolar macrophages, dendritic cells (DCs) and peripheral blood mononuclear cells (PBMCs) [5, 7, 8]. Consistent with these findings, PBMCs from asthmatics have impaired expression of antiviral IFN responsive genes, such as *MxA* and *OAS1,* in response to rhinovirus [5]. Despite mounting evidence that asthmatics have increased susceptibility to respiratory infections through impaired antiviral mechanisms, specific approaches to profile these impairments and link them to increased susceptibility to AETRIs are not generally used. Such approaches would facilitate grouping individuals according to their immune defects and identify novel risk factors for AETRI.

Given the significant morbidity associated with asthma exacerbations and the observation that some individuals have increased susceptibility to AETRI, we sought to characterize the transcriptomic response to single and multiple AETRIs in PBMCs of subjects with asthma using publicly available datasets. We hypothesized that subjects with AETRI exhibit distinct transcriptomic abnormalities in key pathways involved in the immune response to respiratory infections. To test this hypothesis, we identified a transcriptomic signature in PMBCs using an in vitro model of rhinovirus exposure and subsequently used this signature to characterize the transcriptomic response in PBMCs to infection in a cohort of adults with single and multiple AETRIs.

## Methods

### Datasets

We identified two asthma datasets in the NCBI Gene Expression Omnibus (https://www.ncbi.nlm.nih.gov/geo/). The first one was an in vitro dataset of virograms for prediction of predisposition to asthma exacerbations, GSE68479 [9]. Briefly, this study collected PBMCs from 23 children with asthma (ages 6–18) participating in the extension studies following the TREXA and BADGER clinical trials [10, 11]. Their PBMCs were used in an in vitro assay that tested paired PBMCs samples in culture for 24 h with and without human rhinovirus serotype 16. Gene expression data were generated with the affymetrix Human Gene 1.0 ST microarray (Affymetrix, Santa Clara, CA). Additional details on RNA collection and extraction methods are described elsewhere [9].

The second dataset (in vivo) was a large, multicentric study of asthma exacerbations in adults (age ≥ 18 years), GSE19301 [12] .Briefly, PBMCs were collected from subjects with asthma at baseline, during and following an asthma exacerbation. Gene expression data were generated with the affymetrix HG-U133A microarray (Affymetrix, Santa Clara, CA). Additional details on RNA collection and extraction methods are described elsewhere [12]. We divided the in vivo dataset into 3 groups: 1) single AETRI; 2) multiple AETRIs; and 3) single non-infectious asthma exacerbations. The rationale behind the stratification was first, individuals with single and multiple AETRIs during the study period differ in their susceptibility to develop respiratory infections; and second, individuals with single non-infectious asthma exacerbations have different triggers and are more likely to represent a heterogeneous population than the single or multiple AETRIs. Based on the original report by Bjornsdottir et al. the vast majority of AETRIs were viral in origin and confirmed by a single researcher [12]. The rationale to use children and adult PBMCs was derived from our previous demonstration that a gene expression signature in the blood can help in the stratification of asthma subgroups in both children and adults, supporting the presence of transcriptomic similarities in the blood between these two age groups [13].

### Computational analyses

**Fig. 1:**
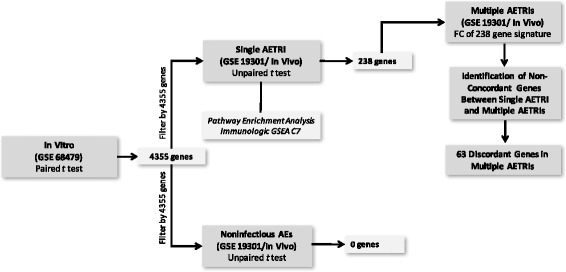
Study design. Abbreviations: *AETRI* asthma exacerbation triggered by respiratory infection, *GSEA* Gene set enrichment analysis, *FC* Fold change, *AEs* Asthma exacerbations

Figure 1 illustrates the workflow used in this study. All microarray CEL files were downloaded for the three datasets. Quality control checks on the microarrays were conducted through removal of low quality samples. Analyses were performed with R software version 3.3.1 (R: A Language and Environment for Statistical Computing. R Foundation for Statistical Computing, Vienna, Austria). Each dataset and subgroup was analyzed using R packages. Normalization was performed with the robust multi-array (RMA) algorithm implementation in the Affy (version 1.52.0) and Oligo (version 1.38.0) packages, and the ComBat function of the sva package (version 3.22.0) was used for batch adjustment [14–16]. Additional statistical and fold change (FC) analyses were performed with Genespring version 12.6 (Agilent Technologies, Santa Clara, CA).

Differences in gene expression for the in vitro dataset, comparing PBMCs from the same subjects cultured with and without rhinovirus, were determined using a paired *t* test, with a false discovery rate (FDR) adjusted *p*-value of < 0.05 and FC ≥1.2. Differentially expressed genes (DEGs) in this analysis were used subsequently to filter the single AETRI and non-infectious asthma exacerbations subgroup analyses. The single AETRI and non-infectious asthma exacerbations analyses compared exacerbation to baseline samples from the same subjects using unpaired *t* tests, based on the difference between the number of baseline samples and exacerbation samples. Significance for these analyses was established at a FC ≥1.1 and FDR-adjusted *p*-value of < 0.1. Pathway enrichment analyses of the pairwise comparisons were performed with MetaCore version 6.27 (Thomson Reuters, New York, NY, USA), analyses with FDR-adjusted *p*-value < 0.05 are reported. Gene set enrichment analysis (GSEA) using the C7 human immunological signatures collection were performed with the GSEA v2.2.0 software using the molecular signatures database (MSigDB) v5.2 (Broad Institute, Cambridge, MA) [17, 18]. The C7 collection includes gene sets that represent cell states and perturbations within the immune system and manually curated signatures of published studies in human and mouse immunology [19]. One thousand permutations based on phenotype were used on the GSEA analyses of normalized matrices for each dataset. Gene set enrichment was considered significant at a qFDR < 0.05. Gender assignment in the in vivo dataset was completed using Staedtler et al., robust and tissue-independent gender-specific transcript biomarkers [20]. Genes with opposite regulation between single and multiple AETRIs (discordant) were identified by comparing FCs in the exacerbation signature identified in single AETRI. Following batch and cell count adjustment with ComBat, baseline comparisons between subjects in the single and multiple AETRIs were performed to identify transcriptomic differences in PBMCs during non-exacerbation periods.

## Results

### Characterization of the transcriptomic response to rhinovirus exposure in PBMCs of subjects with asthma

In order to characterize the transcriptomic profile of PBMC in a model of acute viral infection, we analyzed an existing dataset on gene expression changes in an in vitro model of rhinovirus exposure in PBMCs of children with asthma. PBMCs from 23 donors were used in an in vitro experiment as part of the development of virograms for prediction of predisposition to asthma exacerbations (GEO GSE68479) by Gardeux et al. [9]. A total of 46 samples, representing matched PBMC samples exposed to rhinovirus and culture medium were used. A large number of genes (*n* = 4354; FC ≥ 1.2 and FDR-adjusted *p*-value < 0.05) were identified using this approach (Fig. 1; Additional file 1: Table S1). *CCL8* (MCP-2) was the most abundant transcript in the rhinovirus-exposed PBMCs with a 30-fold increase. *CCL8* is an IFN-inducible chemokine involved in chemotaxis of monocytes, lymphocytes, basophils and eosinophils, and an important component of the antiviral response. [21] Consistent with this finding, several type I interferons (*IFNA* -1,-4,-5,-7,-10,-13,-14,-16,-17 and − 21) and interferon-stimulated genes (ISGs) *IFIT* − 1 and − 2, and *IFI27*, were abundant above a 10-fold increase, confirming the induction of an immune response to rhinovirus (Additional file 1: Table S1). These findings show that the transcriptional profile of PBMCs in a model of rhinovirus exposure is characterized by a robust induction of an IFN-mediated response to rhinovirus. We used this profile as a tool to focus gene expression analyses of PBMC in response to respiratory infections during asthma exacerbations in subsequent experiments.

To characterize the pathways and networks involved in this PBMC-specific response to rhinovirus, we performed pathway enrichment analysis of the differentially expressed genes. The analysis showed a prominent activation of the IL-10 and IL-18 signaling pathways. The IL-10 pathway has been associated with antiviral responses in DCs and regulatory T cells, and found to be elevated in rhinovirus-induced lower respiratory illness [22, 23]. Consistent with the experimental design to test the response to rhinovirus in PBMCs, the gene ontology, network, and disease enrichment analyses showed activation of IFN signaling, antigen presentation and response to infection, respectively (Additional file 1: Table S2). These findings collectively support the induction of the antiviral response to rhinovirus at 24 h in PBMCs from children with asthma and may assist in the identification of immune transcripts involved in the PBMC response to AETRI.

### The PBMC transcriptome in asthma exacerbations has increased type-I IFN activation and discriminates single AETRI from non-infectious asthma exacerbations

In order to validate our observations from the in vitro experiment, we sought to identify similar gene expression changes in PBMCs from subjects in an existing dataset of asthma exacerbations [12]. We subdivided the study population from the in vivo cohort in three groups: single AETRI, multiple AETRIs, and non-infectious asthma exacerbations. Clinical characteristics of the three groups are summarized in Table 1.

**Table 1:** Characteristics of subjects in the in vivo dataset

	Single AETRI	Non-infectious AEs	Multiple AETRIs
	(*N* = 41)	(*N* = 38)	(*N* = 11)
Exacerbation samples (n)	41	38	27
Baseline samples (n)	147	124	32
Female Gender	28 (68%)	22 (58%)	9 (82%)
White Race	36 (88%)	33 (87%)	10 (91%)
BMI, kg/m^2^	27.7 [24.5–31.3]	27.8 [24.7–32.0]	34.2 [27.9–38.7]
GERD	8 (20%)	13 (34%)	6 (55%)
Atopy Status			
Atopic	27 (66%)	25 (66%)	8 (73%)
Non-Atopic	9 (22%)	8 (21%)	1 (9%)
Unknown	5 (12%)	5 (13%)	2 (18%)
Asthma Severity at the time of Exacerbation			
Mild Persistent	5 (12%)	2 (5%)	0 (0%)
Moderate Persistent	20 (49%)	16 (42%)	4 (36%)
Severe Persistent	16 (39%)	20 (53%)	7 (64%)
FEV1 predicted (%)	88 [65–98]	78 [62–87]	62 [45–95]
IgE level, IU/ml	107 [46–224]	117 [53–198]	64 [47–65]
Inhaled CS use	13 (32%)	19 (50%)	10 (91%)
Systemic CS use	15 (37%)	12 (32%)	4 (36%)
Leukotriene receptor antagonist use	15 (37%)	12 (32%)	4 (36%)
Maximum steroid exposure during an exacerbation			
Systemic	13 (32%)	19 (50%)	10 (91%)
Inhaled	27 (66%)	19 (50%)	1 (9%)
Other	1 (2%)	0 (0%)	0 (0%)
Bactin-GAPDH 5′ 3′ ratio exacerbation	0.86 [0.77–0.96]	0.84 [0.78–0.95]	0.86 [0.69–0.93]
Bactin-GAPDH 5′ 3′ ratio baseline	0.85 [0.70–0.93]	0.84 [0.73–0.92]	0.85 [0.73–0.92]
Ratio of M:L whole blood exacerbation	0.19 [0.15–0.32]	0.26 [0.15–0.31]	0.24 [0.20–0.33]
Ratio of M:L whole blood baseline	0.17 [0.13–0.22]	0.20 [0.15–0.26]	0.23 [0.21–0.26]

Data are n (%) or median [25–75 interquartile range] unless otherwise specified

*Definition of* Abbreviations: *AETRI* asthma exacerbation triggered by respiratory infection, *AEs* asthma exacerbations, *BMI* body mass index, *GERD* gastroesophageal reflux disease, *FEV1* forced expiratory volume in 1 s, *IgE* immunoglobulin E, *CS* corticosteroids, *GAPDH* glyceraldehyde-3-phosphate dehydrogenase, *M:L* monocytes to lymphocytes

The single AETRI group consisted of 41 subjects with asthma and a single exacerbation. A total of 41 exacerbation samples and 147 baseline samples (non-exacerbation) that passed quality control checks were used for this analysis. (Figure 1; Table 1) This group was predominantly female (68%), white (88%), non-obese, with non-severe asthma (61%) and the median FEV1 was within normal limits (88% of predicted). Use of inhaled steroids was 66% at the time of exacerbation, with the remaining 34% using systemic steroids.

The non-infectious asthma exacerbations group contained 38 subjects; 38 exacerbation and 124 baseline samples. This group was predominantly female (58%), white (87%), non-obese; with a non-significant higher rate of severe asthma (53%) and similar FEV1 (78% of predicted) compared to single AETRI. The rate of systemic steroid exposure during non-infectious asthma exacerbations (50%) was higher than in single AETRI (32%; *p* < 0.01).

Analyses to identify DEGs between single AETRI and non-infectious asthma exacerbations were filtered by the 4354 genes identified in the in vitro cohort, as described above. In each group we compared exacerbation samples with baseline sample(s) from the same individuals. In single AETRI, we identified 238 DEGs, the majority were upregulated (86%) with modest fold changes (< 2-fold) (Fig. 1; Table 1; Additional file 1: Table S3). A similar analysis in the non-infectious asthma exacerbations group showed no DEGs (Fig. 1). These results indicate that a transcriptomic signature in PBMCs discriminates single AETRIs from non-infectious asthma exacerbations.

**Fig. 2:**
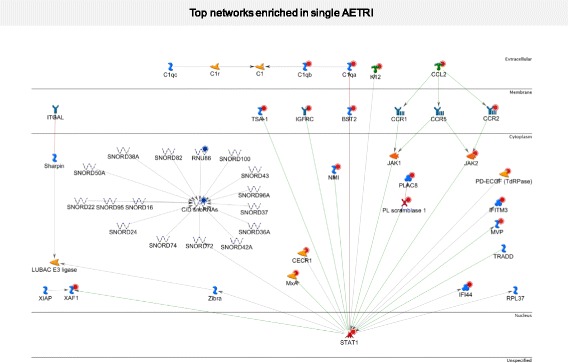
Top network enriched in single AETRI. Several upregulated genes are part of this network including STAT1, JAK2, several interferon induced proteins, CCL2 and CCR2

To characterize the pathways and networks in single AETRI, we performed pathway enrichment analyses with MetaCore in the 238 DEGs. Pathway analyses show upregulation of ISGs and networks involved in the IFN-mediated response to airway infections, including *STAT1* and *JAK2* (Additional file 1: Tables S4 and S5). Moreover, the highest ranked network in single AETRI was also a *STAT1*-dependent network (*p* < 0.001; Fig. 2).

### The transcriptome of activated dendritic cells is enriched in single AETRI

**Fig. 3:**
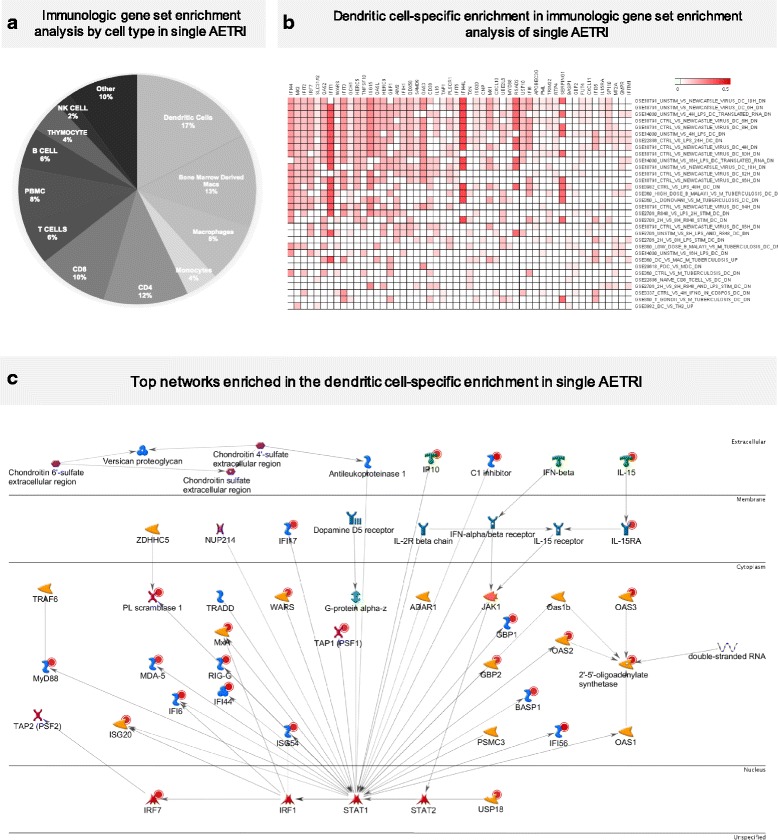
**a** Immunologic gene set enrichment analysis by cell type in single AETRI. This enrichment analysis found a large enrichment (17%) for activated dendritic cells. **b** Dendritic cell-specific enrichment in immunologic gene set enrichment analysis of single AETRI. Characterization of the cell-specific enrichment for dendritic cells was based on upregulation of several interferon-induced genes and IL-15. * This computational method identifies immunologic GSEA pathways of experiments that have similar gene expression characteristics to the experiment being analyzed. The names of those experiments are in the titles of the pathway and as a result they include some description of how they were generated. For example: “DC vs MAC M Tuberculosis up” is telling us that the gene expression profile in our experiment is correlated with upregulated genes in an existing gene expression study that compared DCs vs. macrophages in response to Mycobacterium Tuberculosis. **c** Top network enriched in the dendritic cell-specific enrichment in single AETRI. MyD88, IL-15, IP-10 and several interferon-induced genes are upregulated in the top network of genes enriched in the dendritic cell-specific enrichment

To characterize the immune response signature of single AETRI and its cellular source, we conducted a GSEA using the C7 human immunological signatures in the MSigDB [19]. Using this computational approach, we found significant enrichment for 387 gene signatures in this collection. Figure 3a shows the distribution of cell type enrichment using immunologic GSEA signatures in the PBMC transcriptome of single AETRI. Of these 387 gene signatures, 17% were DC-associated signatures, representing the largest contribution from all cell types. This finding is notable since DCs represent only a small fraction (2%) of the whole PBMC compartment [24]. To characterize this observation further, we generated a correlation heatmap of representative DC-associated genes and pathways enriched in the immunologic GSEA analysis (Fig. 3b shows genes present in > 15% of enriched pathways and pathways with > 15% of these genes in PBMCs of single AETRI). We identified 52 DC-associated genes involved in innate immune and antiviral responses. Importantly, in this DC enrichment subset, the majority of the upregulated transcripts were type I IFN activation and downstream ISGs during single AETRI (Fig. 3c). A similar analysis in non-infectious asthma exacerbations did not show such activation of IFN signaling or enrichment for DC-associated transcripts (not shown). Among the correlated DC datasets in Fig. 3b, GSE29618 showed enrichment for 4 upregulated genes (*OAS3, GBP1, GBP2* and *BASP1*) in myeloid DCs (mDCs) vs. plasmacytoid DCs (pDCs). Of these 4 genes, only *OAS3* and *GBP1* were upregulated during AETRI. These findings suggest enrichment for mDCs but given the limited number of DEGs, it is not possible to discriminate the role of mDCs and pDCs in AETRI. Taken together, these network enrichment analyses and immunologic GSEA point to increased type I IFN activation in single AETRI with a strong DC-associated gene signal. This signature is specific to single AETRI, based on the absence of a similar response in non-infectious asthma exacerbations.

### STAT1 signaling is decreased at the time of exacerbation in multiple AETRIs compared to single AETRI

**Fig. 4:**
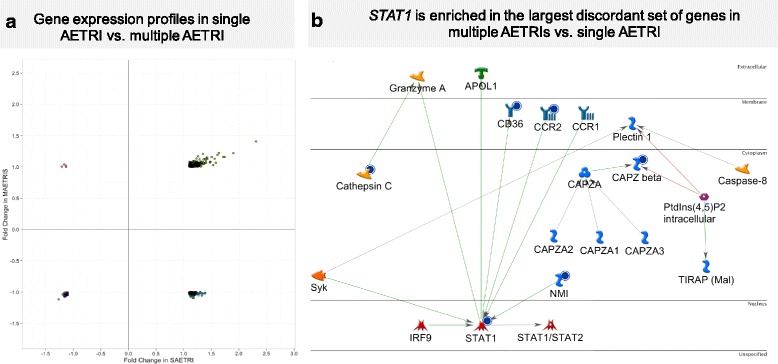
**a** Gene expression profiles in single AETRI vs. multiple AETRIs. This graph represents the fold change relation between single and multiple AETRIs and identifies two discordant subsets of genes (left upper quadrant and right lower quadrant). The large right lower quadrant includes *STAT1*. **b** *STAT1* is enriched in the largest discordant set of genes in multiple AETRIs vs. single AETRI. This *STAT1* network derived from genes in the right lower quadrant of Fig. 4a represents genes that failed to respond in individuals with multiple AETRIs when compared with single AETRI

To identify gene expression changes that differentiate single from multiple AETRIs and may identify predisposition to increased susceptibility to asthma exacerbations, we compared clinical and transcriptomic differences between these groups. The multiple AETRIs group was almost exclusively female (82%), white (91%), obese (BMI = 34.2 [27.9–38.7] kg/m^2^, *p* = 0.03), with high rates of severe asthma (64%) and decreased FEV1 (62% of predicted), Table 1. Systemic steroid exposure was higher during the acute exacerbation than single AETRI (91% vs. 32%; *p* < 0.01). A higher value in the monocyte-to-lymphocyte ratio was seen in multiple AETRIs compared to single AETRI during the acute exacerbation period (0.24 vs. 0.19; *p* = 0.04). To compare the gene expression differences in the AETRI signature (238 DEG), we compared FC differences between single and multiple AETRIs groups (Additional file 1: Table S6). We found an overall positive correlation between FCs in both groups (r2 = 0.44; *p* < 0.01). However, two gene subsets showed prominent differences between single and multiple AETRIs during exacerbations (Fig. 4a; Additional file 1: Table S6). The largest subset consisted of 63 genes that were upregulated in single AETRI but downregulated in multiple AETRIs. The second subset included 4 genes that were upregulated in multiple AETRIs but downregulated in single AETRI (*FBXO16, DYRK2, ID3, PIK3R1*; Additional file 1: Table S6). Given the larger size and potential biologic association with multiple AETRIs we focused on the 63-gene discordant signature. Pathway enrichment analysis showed that the top ranked pathway was the SLE genetic marker-specific pathway in antigen-presenting cells (*p* = 0.01), this pathway includes important elements of IFN signaling (*STAT1*, and IFN-induced with helicase C domain 1-IFIH1 [MDA-5]) (Additional file 1: Table S7). Further analyses demonstrate that several *STAT1* transcription factor networks were enriched in the discordant genes (*p* < 0.001; Additional file 1: Table S8). A network depicting the relationships *between STAT1, CD36, CAPZ beta, NMI, CCR2* is presented in Fig. 4b. This figure provides a better understanding of the direct relation between *STAT1* and other genes in the discordant signature including thrombospondin (CD36), the N-myc, STAT interactor (NMI) [25, 26]. Collectively, these observations identify a type I IFN-associated acute phase signature in subjects with multiple AETRIs that differs from single AETRI. This transcriptional signature is characterized by blunted upregulation of *STAT1* and downstream ISG responses during AETRI, and may underlie the increased susceptibility to multiple AETRIs.

### Multiple AETRIs are associated with increased baseline expression of type I interferons and *STAT1* in PBMCs

**Fig. 5:**
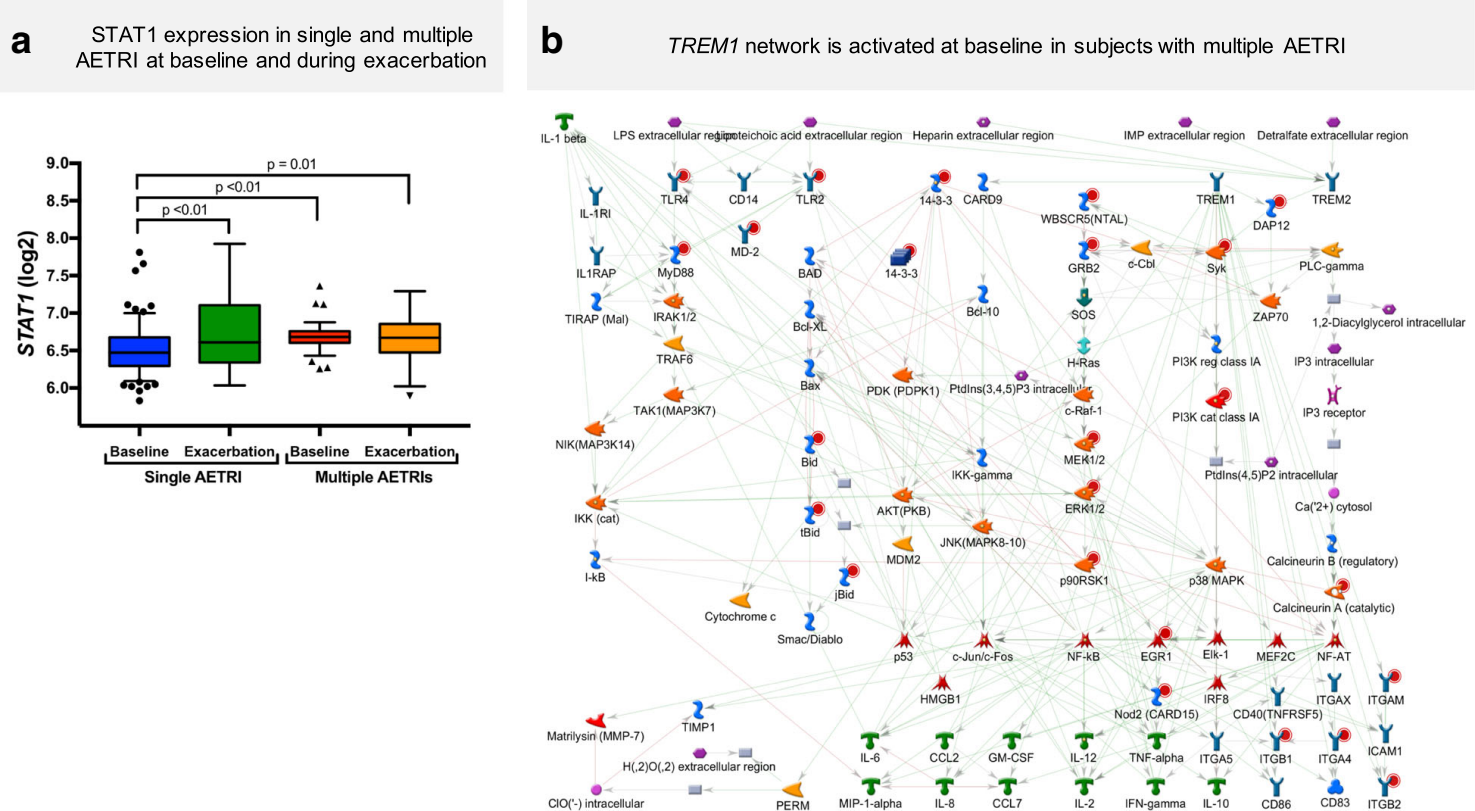
**a** STAT1 expression in single and multiple AETRIs at baseline and during exacerbation. **b** A *TREM1* network is activated at baseline in subjects with multiple AETRIs. This network demonstrates a pro-inflammatory profile at baseline in subjects with multiple AETRIs. Several TLRs, MyD88 and other molecules associated with this TREM1 network are proinflammatory and may underlie the increased susceptibility to multiple AETRIs

Due to the observed differences during the acute exacerbation response between subjects with single and multiple AETRIs, we hypothesized that similar differences could also be present at baseline, identifying a group predisposed to multiple exacerbations. We compared baseline PBMC samples of individuals with single and multiple AETRIs. The difference in the monocyte-to-lymphocyte ratio seen during the asthma exacerbations was maintained at baseline (0.23 vs 0.17; *p* < 0.01). Accordingly, we adjusted our analysis by the monocyte-to-lymphocyte ratio, in addition to batch adjustment. We identified 830 DEGs between these two groups at baseline (Additional file 1: Table S9). *STAT1* was upregulated at baseline in subjects with multiple AETRIs vs. single AETRI (FC = 1.2), contrasting with *STAT1* downregulation seen in the same group during the acute exacerbation period (Fig. 5a). Similarly, several ISGs and IL-15 were present among the upregulated transcripts at baseline in subjects with multiple AETRIs. The upregulation of IL-15 at baseline in multiple AETRIs is similar to the IL-15 upregulation seen in the DC signature during the acute exacerbation period in single AETRI. Enrichment analyses of these DEGs identified that the top two process networks were neutrophil activation and *TREM1* signaling (Additional file 1: Table S10). Figure 5b illustrates changes in the *TREM1* network. *TREM1* is a surface marker of neutrophils and monocytes that is activated during viral infections [27, 28] and a recent report identified that upregulation of *TREM1* in peripheral blood is associated with decreased asthma control [29]. Collectively, these findings indicate the presence of a pro-inflammatory transcriptional pattern at baseline in subjects with multiple AETRIs. Specifically, increased expression of *STAT1*, ISGs, IL-15 and a *TREM1* network.

## Discussion

This study shows that subjects with asthma and multiple AETRIs exhibit a pro-inflammatory PBMC transcriptome at baseline characterized by abnormalities in STAT1 and IL-15 and a dysregulated transcriptional profile during asthma exacerbations. These profiles were identified by contrasting PBMC profiles in three groups of asthmatics with single and multiple AETRIs, and non-infectious asthma exacerbations. Analysis of the PBMC transcriptome was motivated by the non-invasive nature of this technique and availability of a large validation dataset [12]. Our approach used an in vitro model of viral infection to identify relevant transcripts followed by the identification of a specific, single AETRI transcriptional signature, helped separate these exacerbations from non-infectious ones. The single AETRI signature was characterized by activation of type I interferon pathways and genes involved in the immune response to bacterial infections in the airway. In contrast with the single AETRI group, subjects with multiple AETRIs demonstrated a blunted *STAT1*-dependent network and ISG response at the time of an exacerbation despite a higher *STAT1* expression at baseline.

The transcriptional signature in PBMCs during single AETRI was specific as it was absent in subjects with non-infectious asthma exacerbations. We used the DEGs identified in an in vitro model of viral infection to discriminate infectious exacerbations from non-infectious asthma exacerbations, given the significant heterogeneity in the latter and our interest in evaluating elements of the antiviral response in asthma (Fig. 1). The single AETRI signature was enriched for activated DCs, suggesting that DCs contribute substantially to the complex transcriptional response to AETRIs despite accounting for a small fraction of cells in the PBMC compartment. Our study was not able to determine from this data what subtypes of DCs are responsible for this transcriptional enrichment. Further characterization of these DC subsets may yield additional insights into their role in AETRIs. A limitation of our findings was the lack of information on the criteria used to determine AETRIs in the original study [12]. We were not able to obtain additional information regarding these criteria. Based on our two-step study design and available information, the best description of the identified transcriptomic signature in the acute response to AETRIs is biased towards viruses. The ability of this signature to distinguish between infectious and non-infectious triggers of asthma exacerbations needs to be validated prospectively, as it is potentially relevant for clinical practice.

We identified dysfunctional gene expression in the acute exacerbation in subjects with multiple AETRIs. Further enrichment analysis found interferon and *STAT1* signaling impairment. This finding is consistent with previous observations of defective antiviral responses in subjects with asthma [5, 30]. Chen et al. found that in subjects with asthma *STAT1* phosphorylation was influenced by IL-4 [31, 32]. We did not find differences in IL4 expression in PBMCs between the single and multiple AETRIs groups at baseline (not shown), however, *STAT1* expression was higher at baseline in subjects with multiple AETRIs than single AETRI. Accordingly, IL4-independent factors may be responsible for the differences seen in *STAT1* gene expression.

In addition to the increased expression of *STAT1* at baseline, we found upregulation of several cell adhesion molecules, TLRs − 2, − 4, − 5 and − 8, and a *TREM1* network at baseline in subjects with multiple AETRIs. This suggests the presence of an activated, pro-inflammatory state that may lead to increased susceptibility to respiratory infections through defective immune cell signaling or skewing of the response to infections. The pro-inflammatory signature at baseline was associated with decreased induction of antiviral genes during multiple AETRIs, although absolute gene expression changes were similar to single AETRI. A recent study identified an association between *TREM1* in a blood signature and asthma control, suggesting that this gene may be a potential marker of disease activity, it is unknown how TREM1 expression, asthma control and asthma exacerbations are related to each other [29].

IL-15 was upregulated in the acute exacerbation response in single AETRI and at baseline in multiple AETRIs. TLR signaling through MYD88 has been shown to induce IL-15 upregulation and *STAT1* is required for IL-15 induction [33, 34] The differences in subjects with multiple AETRIs at baseline were not maintained during the acute exacerbation period characterized by IL-15 induction in single AETRI (FC = 1.21), but no significant IL-15 upregulation in multiple AETRIs (FC = 1.03). IL-15 deficiency in asthma exacerbations has been previously identified in bronchoalveolar lavage macrophages [35]. In asthma, IL-15 has been associated with regulation of eosinophil viability [36]. Based on these previous observations and our novel finding in multiple AETRIs, the role of IL-15 in asthma appears to be context specific with different roles at baseline and during respiratory infections. We speculate that the increased IL-15 expression at baseline may be associated with increased eosinophil survival and the decreased IL-15 response during the acute exacerbation period may reflect inhibition of IL-15 production due to constant stimulation of IL-15 producing cells at baseline or exhaustion of these cells. These findings of *STAT1* and IL-15 expression in the PBMC transcriptome at baseline in subjects with multiple AETRIs support the presence of a pro-inflammatory state characterized by upregulation of genes involved in monocyte activation and eosinophil survival, leading to increased susceptibility to infectious exacerbations.

Potential mechanisms of increased basal activation of type I interferons, STAT1, and IL-15 may reflect chronic antigenic, including chronic viral infections (HIV, Hepatitis B and C); decreased T-cell inhibitory activity; sustained inflammatory activity leading to T cell exhaustion [37]; and autoimmune diseases where IFN activation is essential for pathogenesis [38]. Plasmacytoid DC contribute to immunopathogenesis through local and systemic effects via release of immune mediators [38]. To exemplify this, pathway enrichment of discordant genes in our analysis identified “antigen-presenting cells in lupus” as the top pathway including (*STAT1* and *MDA5*). Future studies should focus on IFN subclass and mechanisms of immune activation driving AETRI in this cohort.

Several questions remain; do activated monocytes or DCs lead to this phenotype? Or do they mediate their effect through increased activation of other cell types including neutrophils, eosinophils and lymphocytes. It is known that IL-15 functions in a contact-dependent manner and promotes T_H_1 cell-mediated immunity leading to tissue destruction mediated through co-stimulation of effector cytotoxic T cells [39]. An additional question is, do these activated cells localize to the lung, leading to increased susceptibility to multiple AETRIs? we speculate that some of the cell activation differences seen in the PBMC compartment may translate into the lung and may be responsible for the multiple AETRIs phenotype.

The use of a pre-existing gene expression dataset led to certain limitations in our study design and data analysis. The use of a transcriptome-based signature does not take into account protein function and post-translational modifications that play a role on cellular function. Our study is also unable to answer if these transcriptomic responses result from specific genetic variation, comorbidities, pharmacotherapy, or virus-specific effects. The limited virus detection data in the in vivo cohort is of particular importance, as RV, Influenza, Parainfluenza, and other viruses may seasonally overlap, yet have distinct immune responses that condition future immune activation to allergens and infection.

We worked to overcome some of these methodological limitations by using a stringent quality control pipeline, including batch adjustment to decrease the influence of batch and cell differential effects. Assessments of gene expression enrichment in specific cell types would be informative, yet this was not possible in our study as differential cell count information was not available. We also used a rational filtering approach to enhance our ability to identify transcriptional elements involved in the response to infection by using an in vitro experiment of the response to rhinovirus in the same cellular compartment (PBMCs) from subjects with asthma. However, we did not have access to data on specific viruses identified at the time of exacerbation, limiting our ability to discriminate antiviral response patterns associated with distinct viral pathogens. There is a potential for overlap between aeroallergens and respiratory pathogens in asthma exacerbations that could be a confounder for our findings. We lack comprehensive information on T_H_2 profiles or surrogate markers of T_H_2 inflammation in these individuals and are unable to use that adjustment to inform our conclusions. The larger exposure to systemic steroids may have affected the AE response in some individuals; however, the presence of transcriptional differences at baseline suggests that the findings in the acute period are preceded by transcriptional abnormalities at baseline. Finally, each group comparison was assembled so patient samples at baseline served as control to their exacerbation samples. Despite these strategies to decrease the impact of the inherent limitations in this study, we believe that prospective validation of the findings is necessary.

We propose that future characterization of asthma exacerbations requires a more refined approach that includes a better identification of respiratory pathogens leading to AETRIs and a comprehensive search for non-infectious triggers. Transcriptomic profiling at the time of exacerbation and at baseline has the potential to uncover immune response defects to respiratory infections and identify molecular features associated with increased susceptibility to infectious exacerbations. Our work provides evidence of impaired antiviral immunity in asthma and signal a potential mechanism for immune dysregulation at baseline and during exacerbations in patient subsets. The non-invasive nature of the PBMC transcriptome increases the likelihood to obtain a sample and may offer important clues to identify distinct pro-inflammatory states responsible for increased susceptibility to asthma exacerbations.

## Conclusion

Subjects with multiple AETRIs can be differentiated at baseline through non-invasive profiling of circulating PBMCs demonstrating a pro-inflammatory state at baseline, characterized by elevated *STAT1,* ISGs and IL-15 expression, as well as enrichment of an upregulated *TREM1* network. This pro-inflammatory profile may underlie the increased frequency of asthma exacerbations triggered by respiratory infections.

## Additional file

Additional file 1: **Table S1.** In Vivo Transcriptome. **Table S2.** Enrichment In Vitro. **Table S3:** Single AETRI Exp. **Table S4.** Enrichment Single AETRI. **Table S5.** Networks Single AETRI. **Table S6.** Fold Single-MultAETRIs. **Table S7.** Enrichment Discordant. **Table S8.** Network Discordant. **Table S9.** Transcriptome Baseline. **Table S10.** Enrichment Baseline. These tables contain the full reports for gene expression differences between the experimental conditions described in the main text, gene enrichment analyses and network analyses. (XLSX 730 kb)

## Abbreviations

AETRI: Asthma exacerbations triggered by respiratory infection
DCs: Dendritic cells
DEGs: Differentially expressed genes
FC: Fold change
FDR: False discovery rate
GSEA: Gene set enrichment analysis
IFNs: Interferons
MHC: Major histocompatibility complex
MSigDB: Molecular signatures database
PBMCs: Peripheral blood mononuclear cells
RMA: Robust multi array
*TREM1*: triggering receptor expressed on myeloid cells 1
